# Massive Levemir (Long-Acting) Insulin Overdose: Case Report

**DOI:** 10.1155/2012/904841

**Published:** 2012-08-09

**Authors:** Mamatha Oduru, Mahmood Ahmad

**Affiliations:** ^1^St Mary's Hospital, Imperial College Healthcare Trust, London W2 1NY, UK; ^2^Cardiology Department, Tahir Heart Institute, Rabwah, Jhang 35460, Pakistan

## Abstract

A 52-year-old insulin-dependant diabetic man presented to the Emergency Department 2 hours after a deliberate massive overdose of 2100 units of long-acting Levemir insulin and a large quantity of whisky. On initial assessment, his GCS was 3/15 and his capillary blood sugar was 2.6 mmol/L. The patient was given a 50 ml bolus of 50% dextrose, followed by intravenous infusions of both 5% and 10% dextrose. Despite the continuous infusions, he experienced 4 symptomatic hypoglycaemic episodes in the first 12 hours after admission. These were managed with oral glucose, IM glucagon, and further dextrose boluses. Blood electrolytes and pH were monitored throughout. Insulin overdoses are relatively common and often occur with an excess of other drugs or alcohol which can enhance its action. Overdoses can result in persistent hypoglycaemia, liver enzyme derangement, electrolyte abnormalities, and neurological damage. Overall mortality is 2.7% with prognosis poorest in patients who are admitted with decreased Glasgow Coma scale (GCS) 12 hours after overdose.

## 1. Case Presentation

A 52-year-old man was brought to the Emergency Department after a deliberate huge overdose of long-acting Levemir insulin following an argument with his partner. He reported injecting 2100 units at multiple points on his chest, abdomen, and upper thighs. He concurrently ingested three quarters of a bottle of whisky (approximately 450 mL of 40% alcohol). No discarded drug packets were found around him. He was brought to hospital by ambulance two hours after his overdose when his partner found him unconscious.

Significant past medical history consisted of insulin-dependant diabetes mellitus treated with Levemir insulin (18 units once daily) and Metformin (850 mg three times daily). He was in full-time employment and lived with his partner. He was a lifelong nonsmoker and denied any illicit drug use or excessive alcohol consumption. He also denied previous deliberate self-harm or suicide attempts.

On initial emergency department examination, his GCS was 3/15. Physical observations were stable and he was apyrexial. Initial capillary blood glucose was 2.6 mmol/L. Physical examination, including a full neurological assessment, was unremarkable. A normal ECG was recorded.

His initial arterial blood gas on 15L oxygen showed: pH 7.38, p02 32.5, Co_2_ 5.5, BE −0.9, and HCO_3_ 24.2. Bloods on admission were Na 139, K 3.3, Cr 59, BR 6, AST 15, ALP 66, and Alb 44. Ethanol level was 104 mg/L (reference range 0–9 mg/L) with Paracetamol and Salicylate levels within normal range. Subsequent bloods showed a corrected calcium of 2.13, magnesium of 0.75, and phosphate of 1.16.

Initial management on admission (with a persisting GCS of 3/15 and blood sugar levels below 3 mmol/L) was a 5 mL bolus of 50% dextrose. This was followed by an intravenous infusion of 5% dextrose with 40 mmol Potassium chloride. Due to a submaximal response, this was switched to 10% dextrose. He experienced 4 subsequent hypoglycaemic episodes (BM < 2.5 mmol/L) within the first 12 hours of admission. He was given 3 doses of 1 mg Glucagon with intravenous fluids. After 12 hours, intermittent hypoglycaemic episodes persisted. These were managed with Hypostop, a 50% Dextrose bolus, and 1 mg Glucagon. Over the course of the infusions, his GCS gradually improved to 13/15. Throughout treatment, there was close monitoring of potassium, magnesium, and phosphate levels, along with regular arterial blood gases to monitor pH. Insulin and C-peptide levels were not checked on this occasion.

He experienced his last hypoglycaemic episode 41 hours after taking the overdose and dextrose infusions were continued for 62 hours in total. Metformin was restarted 10 hours after stopping the dextrose infusion.

## 2. Discussion

Insulin overdoses are an increasing clinical problem in the Emergency Department [1]; however, reports of massive overdoses in the literature remain scarce. On a PubMed search, we noted multiple case reports of large insulin overdoses the largest of which was 10 000 units of Humulin R [2]. Other overdoses were 800 units of lispro and 3800 units of glargine in a patient [3], 2500 units of NPH insulin in a patient [4] and 750 units regular insulin and 750 units NPH insulin in a patient [5]. A case of 1500 units of overdose of insulin glargine was also reported. In this case the effects of the Insulin glargine lingered for up to 84 hours after dose [6]. 

It should be noted that insulin overdoses can also be because medical staff have mistakenly given a high dose. In one case in Trafford, a nurse gave 60 units which was ten times the patient's prescribed dose resulting in the death of the patient [7].

Insulin overdose is associated with multiple side effects including neurological damage, electrolyte abnormalities (hypokalemia, hypomagnesaemia, and hypophosphatemia), severe hypoglycaemic episodes, and liver enzyme derangement [8]. 42.8% of overdoses involve long-acting insulins and many are accompanied by concurrent use of alcohol and drugs (most commonly Benzodiazepines) [9]. 

Alcohol is a potent inhibitor of gluconeogenesis due to a change in the hepatic redox state caused by the alcohol dehydrogenase reaction, which decreases the free NAD (+)/free NADH ratio [10]. 

Von Mach et al. have shown that half of cases of overdose present within the first six hours after the overdose. 2.7% of insulin overdose patients will have long-term cerebral defects whilst mortality is 2.7%. Prognosis is poorer in patients who are admitted unconscious 12 hours after overdose. Russell et al. have shown that there is a link between depression and insulin overdoses in diabetics as it is a readily available means of attempting suicide [11]. 

## 3. Physiology and Relation to Case

Levemir is a long-acting insulin which delivers a constant level of insulin between meals. The long duration of action of Levemir and concurrent alcohol usage may have contributed to the persistent hypoglycaemic episodes which lasted 41 hours in this case. Alcohol is associated with increased sensitivity to insulin, possibly through an increase in adiponectin and a subsequent decrease in TNF-*α*. Hence, alcohol usage leads to a poorer prognosis in insulin-overdose patients.

Absorption of subcutaneous insulin varies according to the type of insulin, area of body, blood flow in the region, exercise, skin fold thickness and differs between diabetics and nondiabetics. Hypoglycaemia in nondiabetic patients is more severe due to endogenous insulin secretion and insulin antibodies. The multiple sites of insulin administration in this patient may account for his varying and unpredictable blood glucose levels which were difficult to control despite continuous infusions. Surgical excision of insulin depot/subcutaneous injection sites may be effective in reducing glucose supplementation requirements after overdose. It appears to be more useful in patients with long-acting insulin overdose [12]. 

## 4. Management

In any hypoglycaemic patient presenting to the Emergency Department, blood glucose should be routinely checked 15–30 minutes after the start of a dextrose infusion to rule out the possibility of an insulin overdose. If blood glucose is not increasing as expected, the clinician must have a low index of suspicion for possible insulin overdose. Close monitoring is required to quickly identify recurrent hypoglycaemic episodes. Unexpected dips in blood glucose may require further supplementation with oral glucose, 50% dextrose boluses, and IM Glucagon.

As illustrated by this case, these patients may require dextrose infusions for an extended period of time and this requirement is modified by insulin dosage and duration of action. Monitoring serum insulin and C-peptide levels may be useful in patients in whom the diagnosis in unclear. In the case of an exogenous insulin overdose, one would expect high insulin levels and low C-peptide levels. Electrolyte abnormalities, especially hypokalaemia, can occur in the overdose situation and should be closely monitored and replaced as appropriate.

## 5. Conclusion

Emergency Department physicians must be aware of the possibility of insulin overdose in any patient presenting with hypoglycaemia. Once the diagnosis has been established, these patients require very close monitoring due to the high risk of recurrent hypoglycaemia and the need for individual tailoring of treatment between patients.
